# Lying and stepping behaviors around corrective or therapeutic claw trimming

**DOI:** 10.3168/jdsc.2020-0044

**Published:** 2021-06-03

**Authors:** S. Paudyal, J.E. Lombard, P. Melendez, I.N. Roman-Muniz, R.J. Callan, F. Maunsell, P. Pinedo

**Affiliations:** 1 Department of Animal Sciences, Colorado State University, Fort Collins 80523; 2 Veterinary Services, APHIS, USDA, Fort Collins, CO 80526; 3 Department of Population Health, College of Veterinary Medicine, University of Georgia, Athens 30602; 4 Department of Clinical Sciences, College of Veterinary Medicine, Colorado State University, Fort Collins 80523; 5 Department of Large Animal Clinical Sciences, College of Veterinary Medicine, University of Florida, Gainesville 32608

## Abstract

The objectives of this study were to test differences in lying and stepping behaviors in lactating cows with altered gait or other signs of pain in the day preceding either corrective claw trimming (CCT) or therapeutic claw trimming (TCT; d −1), including healthy controls (HC) as a reference group, and to analyze the associations between categories of lying and stepping behaviors at d −1 and subsequent submission to CCT or TCT (d 0). In addition, we characterized variations in lying and stepping behaviors within 7 d relative to CCT or treatment for a specific foot condition (TCT). A total of 310 Holstein cows in a USDA-certified organic dairy herd in northern Colorado were enrolled. Cows were affixed with an accelerometer (IceQube, IceRobotics) below the fetlock of one hind leg at 12 ± 8 d in milk and monitored for 6 mo for daily activity [lying time (LY; min/d), lying bouts (LB; no./d), and steps (ST; no./d)]. Cows with altered gait or other signs of pain were submitted to claw trimming (CT; d 0) and differentiated as receiving only corrective interventions (CCT) or as being treated for a lameness disorder (TCT). Concurrent activity from cows not submitted to CT was considered a reference from HC. Daily averages for each activity within ±7 d relative to CT were compared among the 3 groups using linear mixed models (PROC MIXED of SAS; SAS Institute Inc.). Logistic regression analyses with a logit link function (PROC GLIMMIX of SAS) were used to test the associations between categories (lower quartile or less, interquartile range, and higher quartile or greater) of lying and stepping behaviors (LY, LB, and ST) preceding CT (d −1) and subsequent submission to CCT or TCT (d 0). On d −1, average (± standard error) LY was greater in TCT (631 ± 34 min/d) than in CCT (568 ± 22 min/d) and HC (581 ± 13 min/d). Overall, LB were not statistically different among groups (CCT = 18.7 ± 0.02 bouts/d; TCT = 19.6 ± 1.17 bouts/d; and HC = 19.1 ± 0.43 bouts/d). Daily ST were lower in TCT (1,810 ± 126 steps/d) than in CCT (2,803 ± 63 steps/d) and HC (2,542 ± 103 steps/d). The odds (confidence interval in parentheses) of TCT were greater in cows in the high LY [3.27 (2.47–4.33)] and high LB [2.31 (1.77–3.02)] categories at d −1. The low ST category was associated with increased odds of CCT [3.32 (1.96–5.65)] and TCT [8.48 (6.49–11.1)]. We concluded that, under the current experimental conditions, cows requiring TCT evidenced greater overall LY and lower daily ST the day before CT. However, these differences were not consistent throughout days in milk categories within the lactation. Type of CT was partially associated with category of activity the day preceding CT, and the associations were more evident for TCT. The magnitude of the behavioral changes before and after CT and the recovery times posttreatment to approach HC behavioral levels varied depending on specific conditions.

## Highlights

• Claw and limb disorders are highly prevalent in dairy cows and represent a significant welfare and economic concern.
• Control strategies typically include regular claw trimming and monitoring and treatment of locomotion dysfunctions.
• Changes in lying and stepping behaviors detected by sensor systems could assist in the early detection of cows requiring intervention.
• The magnitude of the behavioral changes of affected cows submitted for claw trimming depended on specific conditions.

## Graphical Abstract

**Figure:**
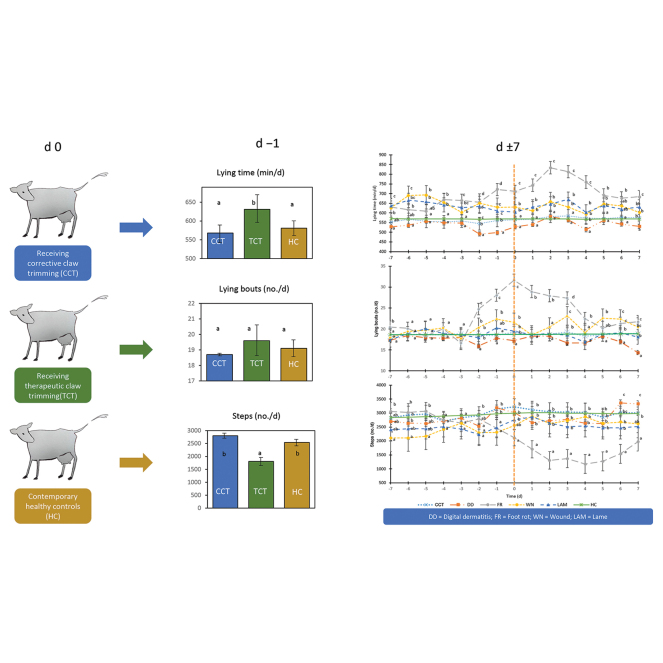
**Summary:** Locomotion disorders are prevalent in dairy cows and represent major welfare and economic concerns. We used sensor technology to test differences in lying and stepping behaviors in lactating cows with altered gait or other signs of pain in the day preceding either corrective or therapeutic claw trimming. Cows requiring therapeutic trimming had greater overall lying time and fewer daily steps the day before treatment. Type of claw trimming was partially associated with activity the day preceding treatment, and these associations were more evident in cows requiring therapeutic trimming. The magnitude of the behavioral changes before and after claw trimming and the recovery times posttreatment varied depending on specific conditions. Monitoring behavioral variables has the potential to detect cows needing interventions for locomotion disorders. However, the detection of specific lameness disorders using activity variables in dairy cows requires further consideration.

Lameness represents a significant welfare and economic concern for dairy systems, and control strategies typically include regular claw trimming (**CT**) and monitoring and treatment of locomotion dysfunctions (Dutton-Regester et al., 2018). In most cases, lameness is an expression of pain and results in a change in a cow's ability to express a normal behavior (Whay and Shearer, 2017). Moreover, a cow's motivation to perform certain behaviors (i.e., walking, lying) may change to alleviate pain and discomfort (Tucker et al., 2021). Sensor systems measuring cow activity have been developed for the identification of lameness disorders, providing an alternative to detection methodologies relying on visual observation (Dutton-Regester et al., 2018; Weigele et al., 2018). Continuous information on behavioral changes also allows for examination of temporary alterations pre- and post-CT and the eventual return to the behavior levels of unaffected mates (Chapinal et al., 2010; Van Hertem et al., 2013).

Our hypothesis was that cows with abnormal gait or other signs of pain would have altered lying and stepping behaviors the day preceding CT and that the magnitude of the changes would be different in cows requiring corrective CT (**CCT**) compared with cows with identifiable foot disorders receiving therapeutic CT (**TCT**). Therefore, the objectives were to test the differences in daily lying time (**LY**) and number of lying bouts (**LB**) and steps (**ST**) preceding either CCT or TCT (d −1) in cows identified with altered gait or other signs of pain and to analyze the associations between categories of lying and stepping behaviors preceding CT (d −1) and subsequent submission to CCT or TCT (d 0). In addition, we characterized variations in LY, LB, and ST within 7 d relative to CCT or treatment for a specific foot condition (TCT). Concurrent activity data from a static group of cows that did not experience any claw disorder during the study period and were not submitted to CT were used as a reference from healthy controls (**HC**).

The study was conducted in a commercial USDA-certified organic dairy herd in northern Colorado milking approximately 3,500 Holstein cows 3 times daily, with a rolling herd average of 8,600 kg/cow (IACUC protocol ID: 17-7665A). The sample size was limited to the number of activity sensors available. A total of 310 Holstein cows (69 primiparous and 241 multiparous) calving between November 2017 and January 2018 were enrolled within 20 DIM and monitored for foot disorders and lameness until June 2018. No criteria other than the calving date were considered in the selection of the study cows. Cows were housed in freestall barns with sand-bedded stalls and had free access to a contiguous dry lot. Average number of cows per pen was 250. Cows were fed a TMR twice a day to meet or exceed the nutritional requirement for a lactating Holstein cow producing 30 kg of milk/d with 3.5% fat and 3.1% true protein (NRC, 2001). During the grazing season (starting on April 23, 2018), cows had access to pasture, which provided at least 30% of the DMI of the total ration.

Cows received CT at least once every 6 mo (approximately at dry-off and again around 180 DIM), and the study herd had a history of lameness associated with digital dermatitis (**DD**) and interdigital dermatitis or foot rot (**FR**), as identified by an experienced claw trimmer working full time on the farm. Cows were also reported with foot wounds and unspecific lameness conditions (**LAM**). Briefly, DD was defined as a painful papillomatous digital lesion, mostly in plantar or palmar skin bordering the interdigital space (Read and Walker, 1998). Foot root was defined as a lesion involving interdigital skin, characterized by fissuring, caseous necrosis of subcutis, and diffuse digital swelling (Read and Walker, 1998). Foot wound included traumatic lesions (cuts or bruises), and LAM was defined as a foot disorder that could not be diagnosed as a specific disease but required an intervention beyond trimming to re-establish appropriate weight bearing and function. The lactating cows walked through a preventive footbath with an acidified 5% copper sulfate solution when exiting the milking parlor twice weekly.

On the day of enrollment (12 ± 8 DIM), cows were affixed with an accelerometer (IceQube, IceRobotics) below the fetlock of one hind leg using a hook-and-loop band. The accelerometer validated by Borchers et al. (2016) provided activity data based on 3-dimensional accelerations collected at 16 Hz. Activity data were provided by the Cowalert system (IceRobotics) summarized in 15-min intervals and downloaded every milking onto a server located in the milking parlor. Study cows were housed in 3 contiguous pens near the milking parlor, containing approximately 250 cows per pen.

The study pens were monitored daily by an experienced claw trimmer to detect cows with altered gait or other signs of pain, such as an arched-back posture or reluctance to bear weight on one or more limbs or feet, which would be equivalent to a lameness score >2 from Sprecher et al. (1997). This target group included subclinical cases (score 3) as well as clinical lameness cases (scores 4 and 5). Monitoring was performed by the claw trimmer located at the exit of the milking parlor alley with full view of cows walking as they returned to their pens. Identified cows were submitted for confirmatory diagnosis and subsequent CT according to the farm's standard operating procedures. Cows submitted to CT were differentiated during examination at the trimming chute as receiving CCT interventions or as being diagnosed with a lameness disorder requiring TCT. Therapeutic CT was considered if the cow needed a curative intervention (blocks, medications, bands). Corrective CT was defined as trimming to correct claw overgrowth and re-establish appropriate weight bearing and function within and between the claws (Shearer and van Amstel, 2001). Study cows were not considered for the scheduled CT at 180 DIM, and a subsequent CT event in the same cow was considered in the analyses as a new case if it occurred after 28 d.

Animals diagnosed with foot disorders were treated according to the farm protocol using therapies approved for use in USDA-certified organic dairies (Pinedo et al., 2017; ECFR, 2021). The main treatment for DD was topical treatment with poultices and emulsions of copper sulfate under a bandage, and cows with FR received iodine and aspirin after wound debridement. Treatment of noninfectious lameness included application of claw blocks to relieve weight bearing on affected hooves (Pinedo et al., 2017). After treatment at the trimming chute, cows were returned to their original pens, which were adjacent and located at a similar distance from the milking parlor.

Daily lying and stepping data were calculated by summing 15-min values reported from 0000 h to 2400 h. Records were exported into spreadsheets (Microsoft Excel; Microsoft Corp.), and cow information was retrieved from PCDART herd management software (Dairy Records Management Systems). Ambient temperature and humidity were continuously measured using Hobo UX100-011 temp/RH 2.5% loggers (Onset Computer Corp.) installed in 6 different points through the farm, and daily temperature-humidity index (**THI**) values were calculated (Kendall et al., 2008). Data sets were organized using Excel spreadsheets, and statistical analyses were completed using SAS (SAS Institute Inc.).

Normality and homoscedasticity of continuous data were graphically evaluated and confirmed before statistical analyses. To facilitate the analyses, DIM were categorized into transition period (≤21 DIM), early lactation (22–150 DIM), and mid lactation (151–212 DIM). Access to pasture, THI category (>68, ≤68), and concurrent estrus were also tested in the models to account for the potential effects of theses variables on activity. The differences in lying and stepping behaviors on d −1 by CT category were tested by ANOVA (PROC MIXED). The final model included CT category, parity category (1, ≥2), concurrent estrus, access to pasture, and THI (Table 1). Logistic regression analyses with a logit link function (PROC GLIMMIX) were used to test the associations between categories of lying and stepping behaviors preceding CT (d −1) and subsequent submission to CCT or TCT (d 0). For this analysis, continuous explanatory variables (LY, LB, ST) were categorized as less than or equal to the lower quartile, interquartile range, and greater than or equal to the higher quartile, respectively, as follows: LY (≤491, 492–662, ≥663 min/d), LB (≤14, 15–21, ≥22 bouts/d), and ST (≤1,437, 1,438–2,067, ≥2,068 steps/d). The final models included activity behavior, parity category, concurrent estrus, access to pasture, THI, and category of DIM (results are reported as adjusted odds ratios in Table 2). Sensitivity and specificity for the ability of LY, LB, and ST on d −1 to predict CCT and TCT were calculated considering values greater than or equal to the higher quartile as positives (PROC FREQ).

**Table 1:** Least squares means ± SE for daily lying time and number of lying bouts and steps on d −1 relative to treatment by claw trimming (CT) category and stage of lactation^1^

Activity^2^	CT category^3^		
	CCT	TCT	HC
Lying time^4^ (min/d)			
Transition period	—	746 ± 64a,b, A–C (4)	632 ± 13a,b, A–C (45)
Early lactation	559 ± 13a,b, A–C (149)	594 ± 24a,b, A–C (52)	575 ± 3a,b, A–C (45)
Mid lactation	574 ± 17a,b, A–C (41)	584 ± 34a,b, A–C (19)	566 ± 3a,b, A–C (45)
Overall	568 ± 22^a^ (190)	631 ± 34^b^ (75)	581 ± 13^a^ (45)
Lying bouts (no./d)			
Transition period	—	22.1 ± 2.99a,b, A–C	19.9 ± 0.64a,b, A–C
Early lactation	18.5 ± 0.63a,b, A–C	14.3 ± 0.38a,b, A–C	18.8 ± 0.23a,b, A–C
Mid lactation	20.3 ± 0.84a,b, A–C	15.9 ± 0.43a,b, A–C	19.2 ± 0.23a,b, A–C
Overall	18.7 ± 0.02^a^	19.6 ± 1.17^a^	19.1 ± 0.43^a^
Steps (no./d)			
Transition period	—	1,745 ± 347a,b, A–C	2,426 ± 135a,b, A–C
Early lactation	2,793 ± 133a,b, A–C	2,079 ± 138a,b, A–C	2,488 ± 48a,b, A–C
Mid lactation	3,480 ± 177a,b, A–C	1,917 ± 379a,b, A–C	3,164 ± 47a,b, A–C
Overall	2,803 ± 63^b^	1,810 ± 126^a^	2,542 ± 103^b^

a,b Different superscripts within a row indicate significant difference between CT categories (*P* < 0.05).

A–C Different superscripts within a column indicate significant difference between DIM categories (*P* < 0.05).

1 Variables included in the model were CT category (CCT, TCT, HC), parity category (*P* < 0.01), concurrent estrus (*P* < 0.001), access to pasture at the time of CT (*P* < 0.001), and temperature-humidity index (>68 vs. ≤68; *P* = 0.23). Two-way interactions were not significant, and they were removed from the model.

2 Transition period: ≤21 DIM; early lactation: 22–150 DIM; mid lactation: 151–212 DIM.

3 CCT = corrective CT (n = 190); TCT = therapeutic CT (n = 75); HC = healthy controls (cows not submitted to CT; n = 45).

4 Value in parentheses is number of cows.

**Table 2:** Adjusted odds ratios (95% CI in parentheses) for corrective claw trimming (CCT; n = 190) and therapeutic claw trimming (TCT; n = 75) by level of activity the day before treatment^1^

Activity^2^	No.^3^	CCT	*P*-value	TCT	*P*-value
Lying time					
High (≥663 min/d)	12,460	0.95 (0.58–1.54)	0.68	3.27 (2.47–4.33)	<0.0001
Medium (492–662 min/d)	25,785	0.87 (0.61–1.26)	0.5	0.93 (0.71–1.25)	0.66
Low (≤491 min/d)	18,414	Referent		Referent	
Lying bouts					
High (≥22/d)	13,797	0.78 (0.47–1.33)	0.47	2.31 (1.77–3.02)	<0.0001
Medium (15–21/d)	27,553	0.87 (0.61–1.26)	0.67	1.19 (0.93–1.53)	0.16
Low (≤14/d)	15,309	Referent		Referent	
Steps					
High (≥2,068/d)	23,322	Referent		Referent	
Medium (1,438–2,067/d)	17,686	1.66 (0.97–2.84)	0.06	0.87 (0.61–1.26)	0.47
Low (≤1,437/d)	15,651	3.32 (1.96–5.65)	<0.0001	8.48 (6.49–11.1)	<0.0001
Parity (1 vs. ≥2)	10,661 vs. 39,377	0.58 (0.38–0.93)	0.02	0.82 (0.68–1.01)	0.04
Concurrent estrus (yes vs. no)	712 vs. 55,947	1.52 (0.46–4.70)	0.48	0.13 (0.11–0.16)	<0.0001
Access to pasture (yes vs. no)	17,087 vs. 39,576	0.46 (0.17–0.94)	0.04	4.72 (3.49–3.36)	<0.0001
THI (>68 vs. ≤68)	2,562 vs. 54,097	0.85 (0.45–1.61)	0.62	1.04 (0.14–7.72)	0.96
DIM^4^					
Transition vs. mid lactation	9,073 vs. 22,042	—	—	0.22 (0.08–0.63)	0.005
Early vs. mid lactation	25,544 vs. 22,042	4.77 (2.09–10.9)	0.0002	1.50 (1.15–1.90)	0.005

1 Lying time, number of bouts, and number of steps were categorized as less than or equal to lower quartile, between lower and higher quartiles, and greater than or equal to higher quartile. Models included activity behavior, parity category (*P* < 0.01), concurrent estrus (*P* < 0.001), access to pasture (*P* < 0.0001), temperature-humidity index (THI; *P* = 0.96), and DIM category (*P* < 0.005). Two-way interactions were not significant, and they were removed from the models.

2 Categories considered to be referents were parity ≥2, concurrent estrus = no, access to pasture = no, THI ≤68, and category of DIM = mid lactation.

3 Number of cow day observations by category.

4 Transition period: ≤21 DIM; early lactation: 22–150 DIM; mid lactation: 151–212 DIM.

Repeated-measures ANOVA (PROC MIXED) were used to assess daily activity behaviors before and after CT (±7 d), and daily comparisons were completed. The model included CT category (considering specific disorders within TCT), time relative to CT, parity category, concurrent estrus, access to pasture, THI, DIM category, and the interaction between CT category and time relative to CT as fixed effects and cow as random effect (Figure 1). The Tukey multiple differences test was used to compare treatments by day where appropriate.

**Figure 1:**
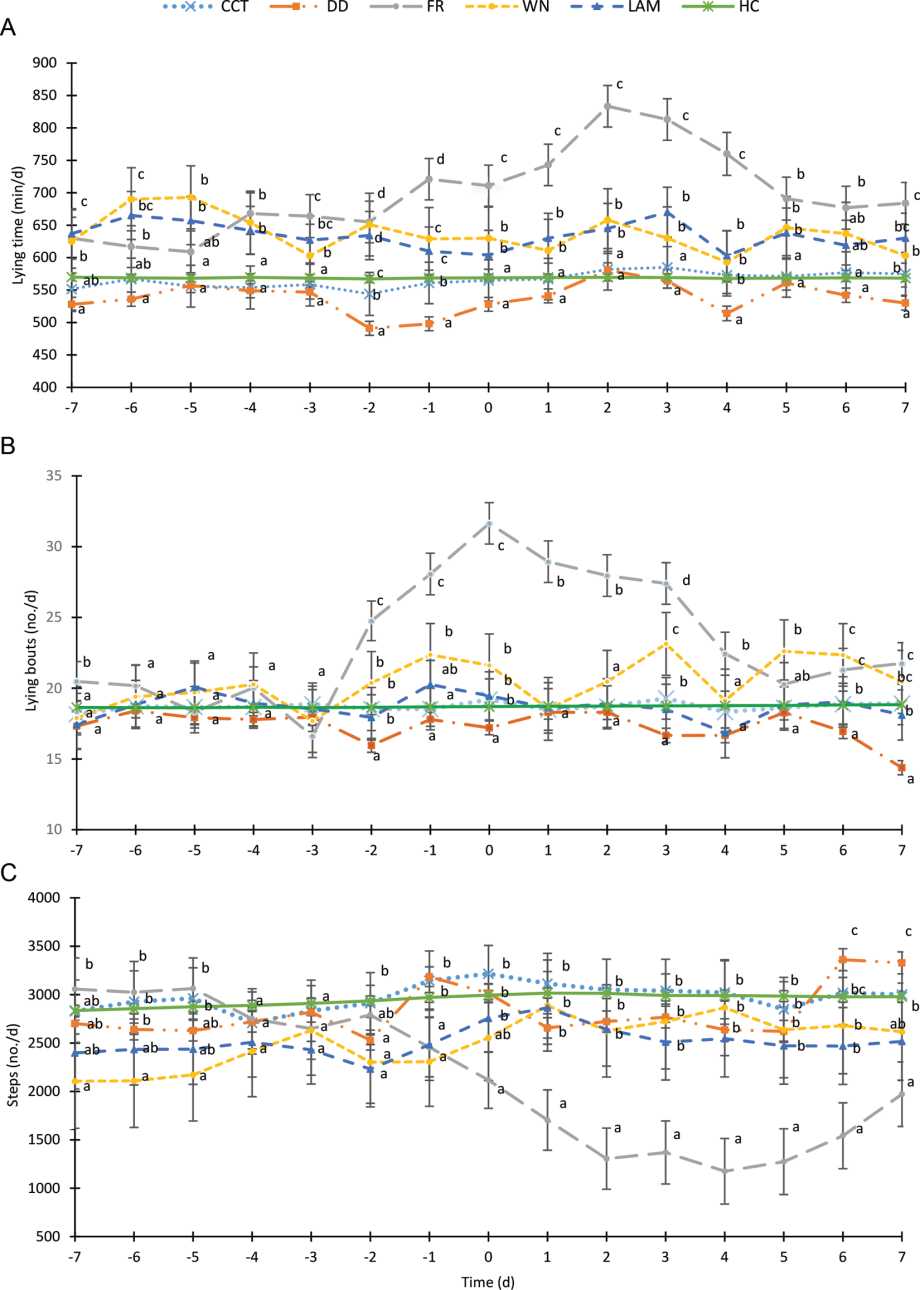
Least squares means (±SE) for daily lying time (panel A) and number of lying bouts (panel B) and steps (panel C) 7 d before and after claw trimming (CT; d 0) in cows receiving corrective trimming or diagnosed and treated for specific lameness disorders. The model included CT category (including specific disorders within TCT), time relative to CT, parity category, concurrent estrus, access to pasture, temperature-humidity index, DIM category, and the interaction between CT category and time relative to CT as fixed effects and cow as random effect. All the other 2-way interactions were not significant, and they were removed from the model. Different letters (a–d) represent significant differences between treatments within each time point (*P* < 0.05). CCT = corrective claw trimming (n = 190); DD = digital dermatitis (n = 21); FR = foot rot (n = 10); WN = foot wound (n = 21); LAM = unspecific lameness (n = 23); HC = not submitted to CT and considered healthy controls (n = 45). The continuous green line is provided as a reference from HC cows.

Pearson correlation coefficients were calculated for all explanatory variables that were considered for inclusion in the multivariable models to detect issues with collinearity, and signs of strong collinearity were not found (−0.32 < r < 0.48). Two-way interactions between all the predictor variables were tested and kept in the final models if *P* ≤ 0.10. For model building, individual explanatory variables were initially tested in univariable models. Variables associated at *P* < 0.25 in the univariable analyses with each outcome variable were included in the multivariable models. The manual backward elimination method was used to remove any variables with *P* > 0.1. Significance and tendency levels were declared at *P* < 0.05 and *P* ≤ 0.1, respectively. As the sample size was limited by the availability of activity sensors and the moderate number of study cows, results should be interpreted with caution.

Overall, 190 cows received CCT and 75 cows developed at least one of the foot disorders considered in the study. Specifically, 21, 10, 21, and 23 cows were diagnosed with DD, FR, foot wounds, and LAM, respectively. Noninfectious foot lesions occurred with low frequency (sole ulcer, n = 3; white line disease, n = 4) and were excluded from the analyses. No cows experienced 2 CT events during the study period. On d −1, LY (min/d) was greater for TCT cows (631 ± 34 min/d; n = 75) than for CCT (568 ± 22 min/d; n = 190) and HC (581 ± 13 min/d; n = 45) cows (*P* < 0.001 and *P* < 0.001, respectively). Overall, LB were not statistically different among groups (CCT = 18.7 ± 0.02; TCT = 19.6 ± 1.17; HC = 19.1 ± 0.43; *P* = 0.21). However, TCT cows had the fewest LB during early and mid lactation. Daily ST were lower in TCT cows (1,810 ± 126) than in CCT (2,803 ± 63) and HC (2,542 ± 103) cows (*P* < 0.001 and *P* < 0.001, respectively; Table 1).

As detailed in Table 2, the odds of TCT were greater (3-fold) for cows in the high LY versus low LY category. For LB, the odds of TCT were more than double for cows in the high bouts versus low bouts category. In addition, the odds of CCT and TCT were significantly greater (3-fold and 8-fold, respectively) for cows in the low ST versus high ST category. Respective sensitivity and specificity (95% CI in parentheses) values for the prediction of CCT were as follows: LY = 22.6% (17.0–29.3%) and 78.0% (77.7–78.4%), LB = 26.8% (20.5–33.7%) and 75.7% (75.3–76.0%), and ST = 17.4 (12.3–23.5%) and 72.3% (71.9–72.8%). Respective sensitivity and specificity values for the prediction of TCT were LY = 37.3% (26.4–49.3%) and 78.0% (77.6–78.5%), LB = 36.0% (25.1–46.9%) and 75.8% (75.3–76.0%), and ST = 42.7% (31.3–54.6%) and 72.4% (72.0–73.0%).

Behavioral variables in the study were affected before and after CT. Figure 1 shows the variations of LT, LB, and ST within ±7 d relative to CT are presented for CCT, specific TCT, and HC.

Considering the painful nature of some of the foot disorders that required TCT (Read and Walker, 1998), the greatest LY and lowest ST at d −1 were expected in this group (Table 1). Most previous reports on the association between locomotion disorders and changes in lying behavior describe increased LY (Singh et al., 1993; Galindo and Broom, 2002; Blackie et al., 2011; Weigele et al., 2018; Tucker et al., 2021). However, some studies also reported no difference (Ito et al., 2010; Yunta et al., 2012) or decreased LY (Cook et al., 2004). In our study we observed greater overall LY in TCT, but when we categorized the animals by lactation stage, the magnitude of the differences combined with our modest sample size did not result in significant differences among CT groups.

We could not establish differences in LB for the overall study period. However, TCT cows had the smallest LB during early and mid lactation. A possible explanation for this behavior in TCT cows is that conditions requiring therapy are likely associated with pain that may reduce the willingness of a cow to stand up once she is lying down (Chapinal et al., 2009). Yunta et al. (2012) observed that lame cows stand up later and lie down earlier after fresh feed is delivered compared with nonlame cows. Moreover, studies with large numbers of cows indicated that in addition to longer lying time, lame cows had fewer, longer lying bouts (Chapinal et al., 2009; Ito et al., 2010; Solano et al., 2016; Westin et al., 2016). Our data partially agreed with these studies, indicating that TCT cows stand up and lie down with less frequency than CCT and HC herd mates, but only during specific times of lactation. Interestingly, LB were increased in FR cows both before and after TCT. In this case, the anticipated effect of discomfort could go in the opposite direction and make cows restless, which increased the number of LB.

As noted previously, our limited sample size and significant variation, evidenced by large standard deviations, had an effect on our ability to detect group differences. For example, a post hoc power analysis for LB indicated that considering the standard deviations and the number of cows for the TCT group (smaller size and greater variation), with power = 80% and confidence = 95%, the difference required to determine significance was 2.3 bouts/d. Although sample size and variation were also limitations for the analyses of LY and ST, the size of the differences in LY and ST between TCT and the other groups was sufficient to establish statistical significance.

In our study, ST was the most consistent behavior across all stages of lactation and was smallest for TCT at all the DIM categories. Notably, at d −1, TCT cows had on average 993 and 732 fewer steps/d than CCT and HC cows. In agreement, Mazrier et al. (2006) reported a reduction in activity (steps/h) for lame cows that ranged from 9 to 68%. In addition, almost half of the lame cows showed a reduction of more than 5% during the 7 to 10 d before clinical signs.

Results from this study indicated that activity behaviors on the day before CT differed for cows having CCT or TCT, which could be an indication of the predictive potential of these behavioral changes. Relative to HC, LY increased by 8.6% in TCT cows and ST decreased by 28%, leading to the possibility of detection of cows needing therapeutic assistance. Notably, this was not the case for CCT cows, which did not show statistical differences from normal cows. These results could be valuable in prioritizing treatment application on the farm and identifying animals that urgently required treatment.

To avoid the effect of management and displacements at the day of CT, we analyzed the associations between categories of activity behaviors during the day preceding CCT or TCT. Our results indicated significance for the association between changes in the 3 behaviors and TCT (odds ratio: 3.27, 2.31, and 8.48 for comparisons between extreme categories of LY, LB, and ST, respectively). On the contrary, only the reduction in ST was associated with subsequent CCT (odds ratio: 3.32; Table 2).

These findings support the potential of automated lameness detection systems that are already in use on commercial farms. However, sensitivity values to predict CT using the suggested categorization of behaviors on d −1 were low, whereas specificity values were moderate. This suggest that the selected cut-off value for the classification of positive and negative cows should be re-examined and that approaches combining multiple variables should be considered.

Notably, our comparison of activity behaviors within ±7 d of CT evidenced some differences in the timing and magnitude of the behavioral changes for distinct claw conditions. Moreover, times to recover and approach levels similar to those of HC were also distinct for some disorders, such as FR (Figure 1). Miguel-Pacheco et al. (2017) identified that recovery from claw conditions depended on severity of the lesion, lesion size, and type of disorder. In a similar study, Miguel-Pacheco et al. (2016) reported that cows receiving both a therapeutic trim and a foot block had greater LY posttreatment compared with cows receiving CCT only. This observation illustrates how a combination of multiple factors may affect cow behavior following treatment. Moreover, activity variations across categories of DIM evidenced in Table 1 should be considered when testing behavioral changes during disease.

In our study, behavioral changes were most evident for FR, where LY, LB, and ST clearly deviated from CCT and normal cows in a large magnitude and for extended time (Figure 1). Specifically, cows affected by FR showed early increments in LB. As this behavior was not part of the claw trimmer assessment, these changes were missed and submission to CT was delayed. In addition, FR cows had decreased ST until the end of the 7-d observation period, which could relate to pain, greater extent of affected tissue, or the limited options for effective treatment under organic certification (Pinedo et al., 2017). It is important to acknowledge that these distinctive behavioral changes identified in FR could be different in conventional farms, where antibiotic use is permitted. This prohibition limits the treatment efficacy for clearing infection, increasing the duration of infection and the number of infectious individuals facilitating transmission, which ultimately results in greater prevalence of this disorder (Pinedo et al., 2017).

We concluded that, under the current experimental conditions, cows requiring TCT evidenced greater overall LY and lower daily ST the day before CT. However, these differences were not consistent throughout lactation periods. Type of CT was partially associated with category of activity the day preceding CT, and the associations were more evident for TCT. The magnitude of the behavioral changes before and after CT and the recovery times required posttreatment to approach HC behavioral levels varied depending on specific conditions.

Monitoring behavioral variables has the potential to detect cows needing interventions for locomotion disorders. However, the detection of specific lameness disorders using activity variables in dairy cows requires further consideration. Future research may be warranted to validate these variables and explore the use of combinations of multiple behaviors.
